# Supplementary material for: Surface Microbiome Profiling of Dental Elevators Using Third‐Generation Sequencing: Implications for Infection Control in Dental Practice
Source: Microbiologyopen. 2025 Dec 8;14(6):e70178. doi: 10.1002/mbo3.70178 (PMC12685768; doi:10.1002/mbo3.70178)
Supplement: Supplementary file 1 — customer_backup. [file MBO3-14-e70178-s001.zip › customer_backup/customer_backup/function_analysis/BugBase/treat/otu_contributions/genus/treat.genus.Anaerobic.pdf]

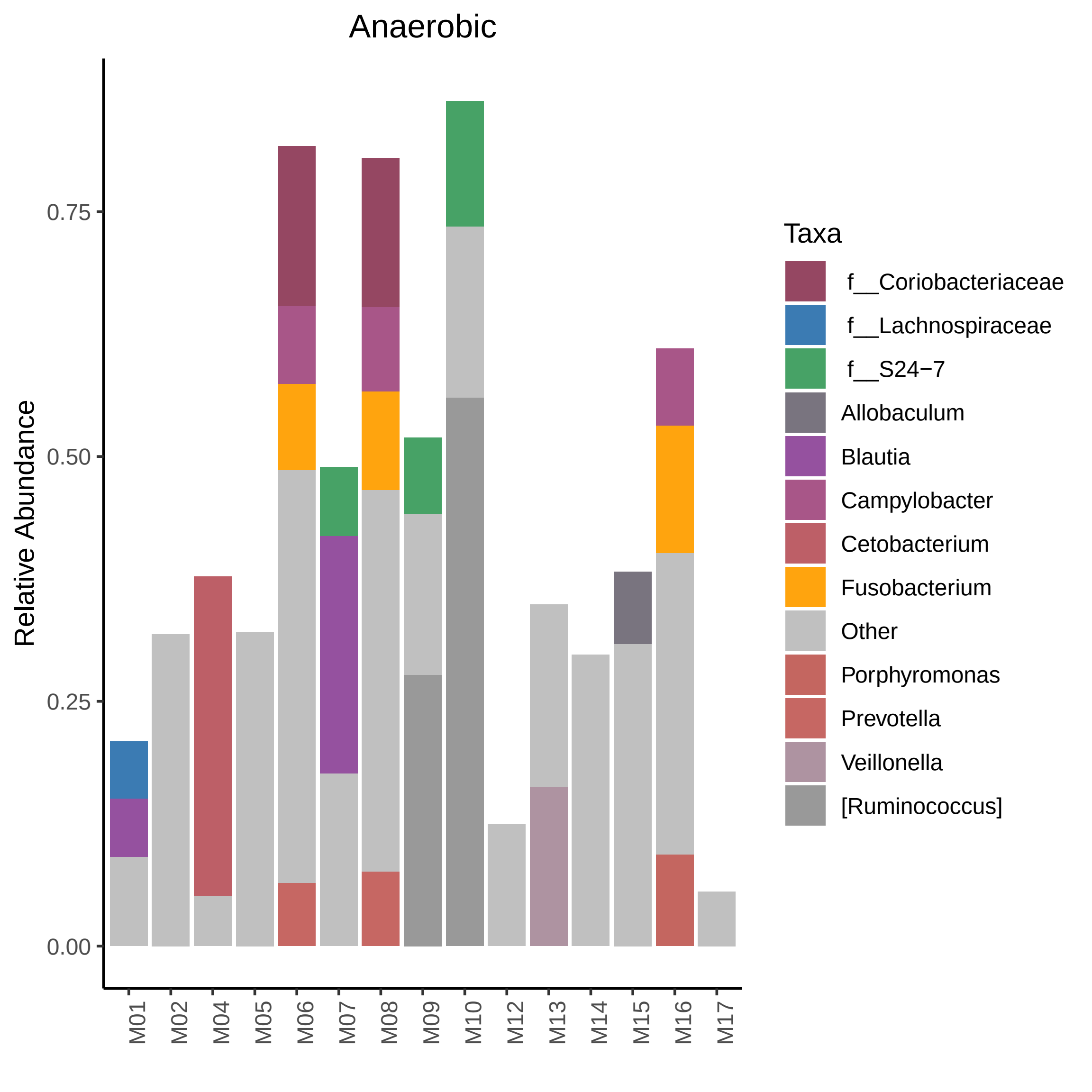
Anaerobic
Relative Abundance
0.00
0.25
0.50
0.75
M01
M02
M04
M05
M06
M07
M08
M09
M10
M12
M13
M14
M15
M16
M17
Taxa
f__Coriobacteriaceae
f__Lachnospiraceae
f__S24−7
Allobaculum
Blautia
Campylobacter
Cetobacterium
Fusobacterium
Other
Porphyromonas
Prevotella
Veillonella
[Ruminococcus]
